# Heterogeneous Mechanisms of Secondary Resistance and Clonal Selection in Sarcoma during Treatment with Nutlin

**DOI:** 10.1371/journal.pone.0137794

**Published:** 2015-10-01

**Authors:** Audrey Laroche, Kevin Tran-Cong, Vanessa Chaire, Pauline Lagarde, Isabelle Hostein, Jean-Michel Coindre, Frederic Chibon, Agnes Neuville, Tom Lesluyes, Carlo Lucchesi, Antoine Italiano

**Affiliations:** 1 INSERM U916, Institut Bergonié, Bordeaux, France; 2 Sarcoma Unit, Institut Bergonié, Bordeaux, France; 3 Pathology Department, Institut Bergonié, Bordeaux, France; Rush University Medical Center, UNITED STATES

## Abstract

Nutlin inhibits TP53-MDM2 interaction and is under investigation in soft-tissue sarcomas (STS) and other malignancies. Molecular mechanisms of secondary resistance to nutlin in STS are unknown. We performed whole-transcriptome sequencing (RNA-seq) on three pretreatment and secondary resistant STS cell lines selected based on their high primary sensitivity to nutlin. Our data identified a subset of cancer gene mutations and ploidy variations that were positively selected following treatment, including TP53 mutations in 2 out of 3 resistant cell lines. Further, secondary resistance to nutlin was associated with deregulation of apoptosis-related genes and marked productive autophagy, the inhibition of which resulted in significant restoration of nutlin-induced cell death. Collectively, our findings argue that secondary resistance to nutlin in STS involved heterogeneous mechanisms resulting from clonal evolution and several biological pathways. Alternative dosing regimens and combination with other targeted agents are needed to achieve successful development of nutlin in the clinical setting.

## Introduction

The tumor suppressor TP53 plays a crucial role in protection from malignant tumor development. It is a transcription factor which is activated following stress and regulates multiple downstream genes involved in cell cycle control, apoptosis, DNA repair, and senescence [1]. In non-stressed cells, the level of T53 is controlled tightly by MDM2 (murine double minute 2). MDM2 regulates p53 through a negative-feedback loop. When the nuclear TP53 level is elevated, it activates the transcription of the *MDM2* gene. In turn, MDM2 binds to TP53 and blocks its transactivation domain. MDM2 also serves as a TP53 ubiquitin ligase that targets TP53 for ubiquitin-dependent degradation in the proteasome [2].

Treatment of cancer cells expressing wild-type TP53 with TP53-MDM2 interaction antagonists should result in the concurrent transcriptional activation of TP53 downstream genes, cell cycle arrest, and apoptosis.

Recently, a class of imidazoline compounds has been identified as potent and selective inhibitors of the TP53-MDM2 interaction [3]. These molecules, termed nutlins, interact specifically with the TP53-binding pocket of MDM2 and thus release TP53 from negative control. Treatment of cancer cells expressing wild type *TP53* with nutlins stabilizes TP53 and activates the TP53 pathway leading to activation of TP53 target genes, cell cycle arrest, apoptosis and/or senescence.

Soft-tissue sarcomas (STS) represent a heterogeneous group of rare tumors including more than 70 different histological subtypes [4]. Many of the STS subtypes probably have specific mechanisms of oncogenesis and may therefore be especially sensitive to appropriate systemic treatments. The identification of new therapies for STS patients is of crucial importance as 30% to 40% of patients with STS will develop metastatic disease [5]. Once metastases are detected, the treatment is mainly based on palliative chemotherapy and median survival of patients in this setting is about 12–18 months [6]. Scores of new agents are in development as cancer therapeutics. Unfortunately, only a fraction of these new agents can be systematically evaluated in soft-tissue sarcomas, largely because of the rarity of pre-clinical models. For instance, no sarcoma cell line is included in the NCI-60 DTP Human Tumor Cell Line Panel. This selection process of potential candidate agents is critical to future progress in curing this rare cancer.

*MDM2* is overexpressed in about 20% of STS including liposarcomas, synovial sarcomas, and leiomyosarcomas [7]. Well-differentiated/dedifferentiated liposarcomas (WDLPS/DDLPS) are one of the most frequent subtypes of STS and are characterized by a specific amplification of the *MDM2* gene [8]. There are only limited pre-clinical data regarding the anti-tumor activity of nutlins in sarcomas [9–11]. Most reports have been based on bone sarcoma and not on STS models. We have recently shown that the MDM2 antagonist activates the TP53 pathway and decreases cell proliferation in patients with TP53-wild type and other solid tumors with or without *MDM2* amplification, and that this was associated with long term disease in about 20% of sarcoma patients included in phase I trials and treated with nutlins [12, 13]. However, all patients who initially benefit from nutlin eventually develop resistance within 6 months to two years after treatment onset [13].

Clonal heterogeneity is a crucial issue for development of personalized cancer medicine [14, 15]. How STS genomic heterogeneity under nutlin selective pressure contributes to acquired resistance is unknown. We reasoned that deep sequencing and bioinformatics could be used to screen the genome of nutlin-resistant cells for clonal genetic aberrations and alterations of biological pathways involved in secondary resistance.

## Material and Methods

### Cells and cell culture

All the STS cell lines used in this study were derived from human surgical specimens of STS in the laboratory of Pr. Jean-Michel Coindre and Dr Frédéric Chibon (Institut Bergonié, Bordeaux, France), and after obtaining written informed patient consent (S1 Table) and Institut Bergonié IRB approval. Each cell line was characterized by array comparative genomic hybridization every 10 replicates to verify that its genomic profile was still representative of the originating tumor sample. The IB111, IB115 and IB128 cell lines used in this study were derived from patients suffering from dedifferentiated liposarcoma (IB111, and IB115) and from extra-skeletal osteosarcoma (IB128). Cells were grown in RPMI medium 1640 (Sigma Life Technologies, Saint Louis, MO) in the presence of 10% fetal calf serum (Dutscher, France) in flasks. They were maintained at 37°C in a humidified atmosphere containing 5% CO_2_.

### Reagents

RG-7388 was supplied by Roche (Roche Pharma Research & Early Development, Basel, Switzerland); doxorubicin was supplied by Accord Healthcare (Lille, France) and gemcitabine by Fresenius Kabi (Sèvres, France).

### Cell viability

Nutlin effects on cell viability were investigated using the MTT assay [3-(4,5-dimethylthiazol-2-yl)-2,5-diphenyl tetrazolium bromide] (Sigma-Aldrich Chimie, Saint-Quentin-Fallavier, France) as an indicator of metabolically-active cells [16]. Known number (2000 or 3000) of STS cells was transferred into 96-well plates incubated for 24 h before addition of test compound. Cells were then exposed for 72 h at 37°C to an increasing concentration range of RG7388, for 48 h to doxorubicine or gemcitabine, MTT at the final concentration of 0.5mg/ml was added and, following incubation for 3h, Formazan crystals were dissolved in DMSO. Absorbance of the colored solution was measured on a microplate-photometre (Bio-Tek Instruments, Colmar, France) using a test wavelength of 570 nm and a reference wavelength of 630 nm. The concentration of substance required for 50% growth inhibition (IC50) was estimated with the Graphpad Prism software (GraphPad software INC, San Diego, USA).

### Cell cycle analysis

Cell cycle distribution of sensitive and resistant cell lines was studied by DNA content by fluorescence-activated cells sorting and analyzed using the Cell Quest Pro software (BD Biosciences, San Jose, USA). After 48h of treatment with two different concentrations of RG7388, cells were centrifuged at 1500g for 5 min and washed twice with PBS. Cells were then fixed with 70% ethanol at 4°C overnight. Ethanol was removed and cells washed twice with PBS. 300μl of a propidium iodide, ribonuclease-containing solution were added to cells and then analyzed by FACS. Data were analyzed with FlowJo v.7.6.3. software and results expressed in terms of percentage of cells in a given phase of cycle.

### Apoptosis

The mitochondrial membrane depolarization (ΔΨm) was measured by tetramethyl-rhodamine ethyl staining (TMRME). 5000 cells were seeded in 96-well plates, incubated 24h before adding an increasing concentration of RG7388 over 48h. Cells were loaded with TMRM (200nM) for 30 min at 37°C and then trypsinized and harvested in saline extracellular solution. Cells were then analyzed on a FAC scan flow cytometer (BD biosciences, San Jose, USA).

For apoptosis assessment, 1.105 cells were seeded in 6-wells plates and after 24h cells were treated by Nutlin for 72hr and exposed to FITC-Annexin and propidium iodide (PI) according to the manufacturer’s protocol (BD Biosciences, Erembodegem, Belgium). It allows distinguishing annexin V positive cells in early apoptosis, versus annexin V and PI positives cells in late apoptosis. Cells were analyzed in cytomerty using FL1 Annexin-V, whereas FL2 was used for Propidium iodide.

Flow cytometry (FACScan; BD Biosciences) data were analyzed with FlowJo v.7.6.3. software.

### Western blotting

Cells were trypsinized, washed in cold phosphate buffered saline (PBS) and lysed by 3 cycles of freezing in liquid azote and thawing in ice as described by Tansey [17]. Cellular extracts were then centrifuged at 13000rpm at 4°C for 10 min and supernatant was incubated with benzonase. 30μg of protein extract was separated on SDS-polyacrylamide gel and transferred to 0.45μM of PVDF membrane in blotting buffer containing 20% of ethanol. The antibodies used were: anti-P53 (1: 200, Santa Cruz sc-126); anti-MDM2 (1: 500, Calbiochem IF2); anti-P21 (1: 33, Calbiochem); anti-LC3-IIB (1: 1000, Sigma-Aldrich); and Anti-GAPDH (1:200, Santa-Cruz Biotechnology). All secondary HRP antibodies were from Dutsher, Brumath, France. Blots were visualized by ECL (Dutsher, Brumath, France).

### Confocal microscopy

Cells were seeded on coverslips and treated with RG-7388 for 72 hours. Slides were then washed twice with PBS, fixed in formaldehyde 4% and incubated with anti-LC3IIB monoclonal antibody (Sigma Aldrich, Saint Louis, USA) overnight, and then with a goat anti-rabbit Alexa fluor 488 antibody (Invitrogen, Paisley, United Kingdom). Slides were then counterstained by 4,6-diamidino-2-phenylindole (Hoechst).

### RnaSeq protocols for Illumina HiSeq2000 NGS

RnaSeq was sequenced by the Centro nacional de análisis genómico (Barcelona, Spain) using standard Illumina (Illumina Inc., 9885 Towne Centre Drive, San Diego, CA, USA) protocols. Briefly, the library from total RNA was prepared using the TruSeq™ Stranded mRNA Sample Preparation kit (Illumina Inc.) according to manufacturer’s protocol. 1μg of total RNA was used for poly-A based mRNA enrichment selection using oligo-dT magnetic beads followed by fragmentation by divalent cations at an elevated temperature resulting into fragments of 80-450nt, with the major peak at 160nt. First strand cDNA synthesis by random hexamers and reverse transcriptase was followed by the second strand cDNA synthesis, performed in the presence of dUTP instead of dTTP. Blunt-ended double stranded cDNA was 3´adenylated and the 3´-“T” nucleotide at the Illumina indexed adapters was used for the adapters’ ligation. The ligation product was amplified with 15 cycles of PCR.

Each library was sequenced using TruSeq SBS Kit v3-HS, in paired end mode with the read length 2x101bp. We generated 135 million minimally-paired end reads for each sample run in one sequencing lane on HiSeq2000 (Illumina, Inc) following the manufacturer’s protocol. Images analysis, base calling and quality scoring of the run were processed using the manufacturer’s software Real Time Analysis (RTA 1.13.48) and followed by generation of FASTQ sequence files by CASAVA.

### NGS RnaSeq sequence alignment and quality control pipeline

Raw RnaSeq sequences where quality controlled using a set of published tools in order to produce curated reads. Briefly, the package sickle [18] was used to trim raw reads of the 5’ and 3’ low quality bases (phred cut off 20, max trim size 30nc, package SeqPrep (St John GitHub, https://github.com/jstjohn/SeqPrep) was used to remove sequencing adaptors from the raw reads. The application of these standard packages revealed a proportion of RNA fragments of smaller size, whose R1 and R2 paired end reads were overlapping. To keep exploiting these fragments without biasing the count of reads in reads overlapping regions, we developed a home-made script (awk language) that merged the overlapping R1 and R2 and split it into new non-overlapping read sequences.

Curated read sequences were then aligned using TopHat2 [19] on both genome and transcriptome with fragment size 175 +/- 70. Following, we applied strict quality control of aligned reads, by filtering the reads whose alignment score was lower than 20 (samtools) and removing the reads with identical align starting positions that were possibly caused by PCR amplification during the creation of the RnaSeq libraries (picard MarkDuplicates at http://broadinstitute.github.io/picard/).

The statistics of the number of raw, curated and aligned sequences and curated aligned sequences was that created with a home-made tool (bash, awk)

### NGS allelic variant detection and annotation pipeline

Positions in the transcriptome showing at least one alternative allele were detected via samtools mpileup [20] taking care to filter out bases with less than 20 as phred quality score. Variant Calling format information was created via bcftools view (v 0.1.19) of the samtools suite.

Positions detected as variant in several samples (sensitive and resistance cell lines) were merged using a homemade script (bash awk).

Variant positions were annotated using the Annovar tool([21]. Briefly, Annovar determines the type of genomic location of the variant for example exonic, intergenic, and its class (e.g. missense, nonsense), its presence in population genetics databases dbSNP [22] and 1000g database [23] and somatic variants (COSMIC, http://cancer.sanger.ac.uk/cancergenome/projects/cosmic). For variants in coding positions the impact of the aminoacid change in the protein domains is reported via the use of Sift [24] and PolyPhen2 [25] databases.

We obtained on average 135M and 120M Paired End reads respectively after sequencing and post-alignment quality controls. As a starting point of our analysis we produced a table, based on post-alignment controlled reads, collecting all genetic variants (either polymorphic or not, single nucleotides or Indels) found in sequences in at least one of the sensitive or resistant samples, of any of the three cell lines. The table resumes the variants that present at least 1 alternative allele counts in any sample, each base having sequencing quality (phred score) of at least 20, without additional filters. The table, containing 285,999 variants (herein called Merged Variant Table) allows checking the alternative and reference allele counts at a position, in any of the sequenced samples.

We extracted from the Merged Variant table the variants detected in sensitive and resistant cells. We then included in the analysis only variants whose genotype was estimated as a non-homozygous reference in both samples, with more than 10 reads at the variant position, localized in coding regions and of non-synonymous type. We included all variants whose positions were reported in the COSMIC database (http://cancer.sanger.ac.uk/cancergenome/projects/cosmic) and whose alternative allele frequency (AF) in the Caucasian population (CEU), as reported in 1000g, was lower than 5%. If a variant was neither reported in 1000g CEU nor dbSNP, it was included only if either SIFT or Polyphen2 HDIV functional interpretations were reported as “deleterious” for the protein. If a variant was not reported in 1000g CEU but reported in dbSNP, it was included only if both SIFT or Polyphen2 HDIV functional interpretations were reported as “deleterious” for the protein. Also, if a variation was located in a region of the genome that is annotated as Segmental Duplication [26], then it was not reported. In order to distinguish variants that were shared between sensitive and resistant cells, we tested whether the Alternative Allele Ratios (AAR) were statistically different between the two conditions (Fisher test *P*< = 10–3, and difference between AAR < = 0.1). Variants whose differences in AAR were not significant and superior to 0.30 in both conditions were associated to the ancestral primary tumor clone, while the other variants identified the differences between the clones sensitive and secondary resistant to RG7388.

### Detection of genomic ploidy and Differential Gene Expression/IPA

RnaSeq was used to estimate the ploidy of the tumor samples, where the term ploidy defines the total number of parental alleles in large genomic regions (see S1 Methods). The RnaSeq Differential Gene Expression between two NGS samples was calculated using the methodology described in S1 Methods and implemented in R language. The selection criteria for detection of Differentially Expressed Genes were based on a Fold Change greater or equal to 2.5 and an adjusted p-value of less than or equal to 1% (Benjamini Hochberg adjustment). Data were analyzed through the use of QIAGEN’s Ingenuity®Pathway Analysis (IPA®, QIAGEN Redwood City,www.qiagen.com/ingenuity) in order to predict the interactions between the genes and the potential effects on cellular pathways and networks.

## Results

### RG7388 activates the TP53 pathway, induces significant proliferation inhibition, cell-cycle arrest and apoptosis in MDM2-amplified and non-amplified STS

**Fig 1:**
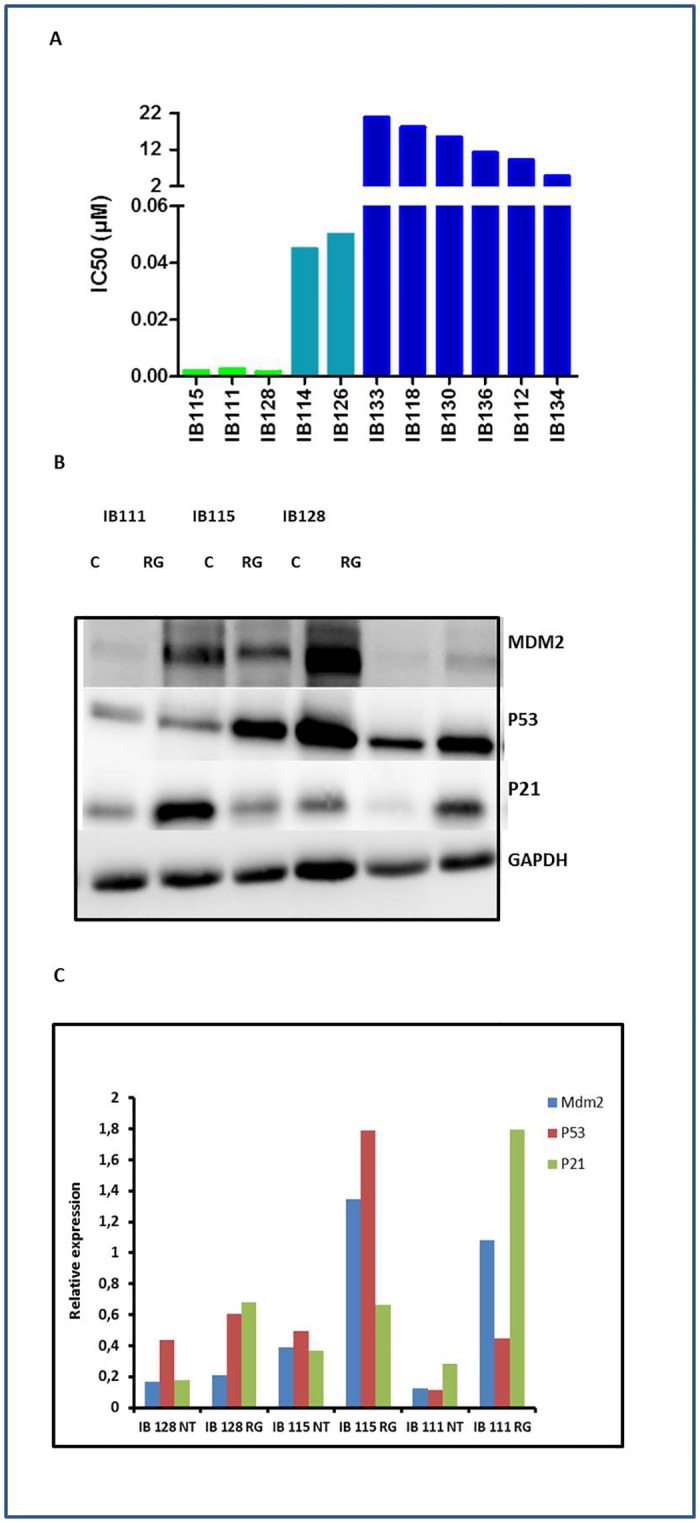
Antiproliferative activity of RG7388 (A) and activation of the p53 (B, C) in human soft-tissue sarcoma (STS) cell lines. (A) IC 50 (μM) of RG-7388 for 11 STS cells, IB111, IB115, IB128, IB114 and IB126 are P53 wild type, the other cell lines are P53-mutated. The experiments presented are representative of at least 3 experiments. Immunoblots are represented on the left (B) and densitometry of the immunoblots on the right (C). Sensitive cells untreated (NT) or exposed to IC50 of RG-7388 (RG) were immunoblotted for MDM2, TP53 and P21 expression.

As predicted by the mechanistic model of TP53 regulation, the nutlin compound RG7388 inhibited significantly the proliferation of 5 out of 11 STS cell lines with no TP53 mutations as assessed by Sanger sequencing (IC50: 2–50 nM), but not of the 6 out of 11 cell lines with TP53 mutations (Fig 1A). The most sensitive cell lines were the DDLPS cell lines IB111 and IB115 characterized by an amplification of the MDM2 gene and the extraskeletal osteosarcoma cell line IB128 characterized with no alteration of the *MDM2* gene copy numbers. In agreement with the mechanism of action of nutlins, treatment of wild-type TP53 (Sanger sequencing) STS cell lines with RG7388 showed an accumulation of the TP53 protein and its targets, P21 and MDM2, as revealed by Western blotting (Fig 1B and 1C).

**Fig 2:**
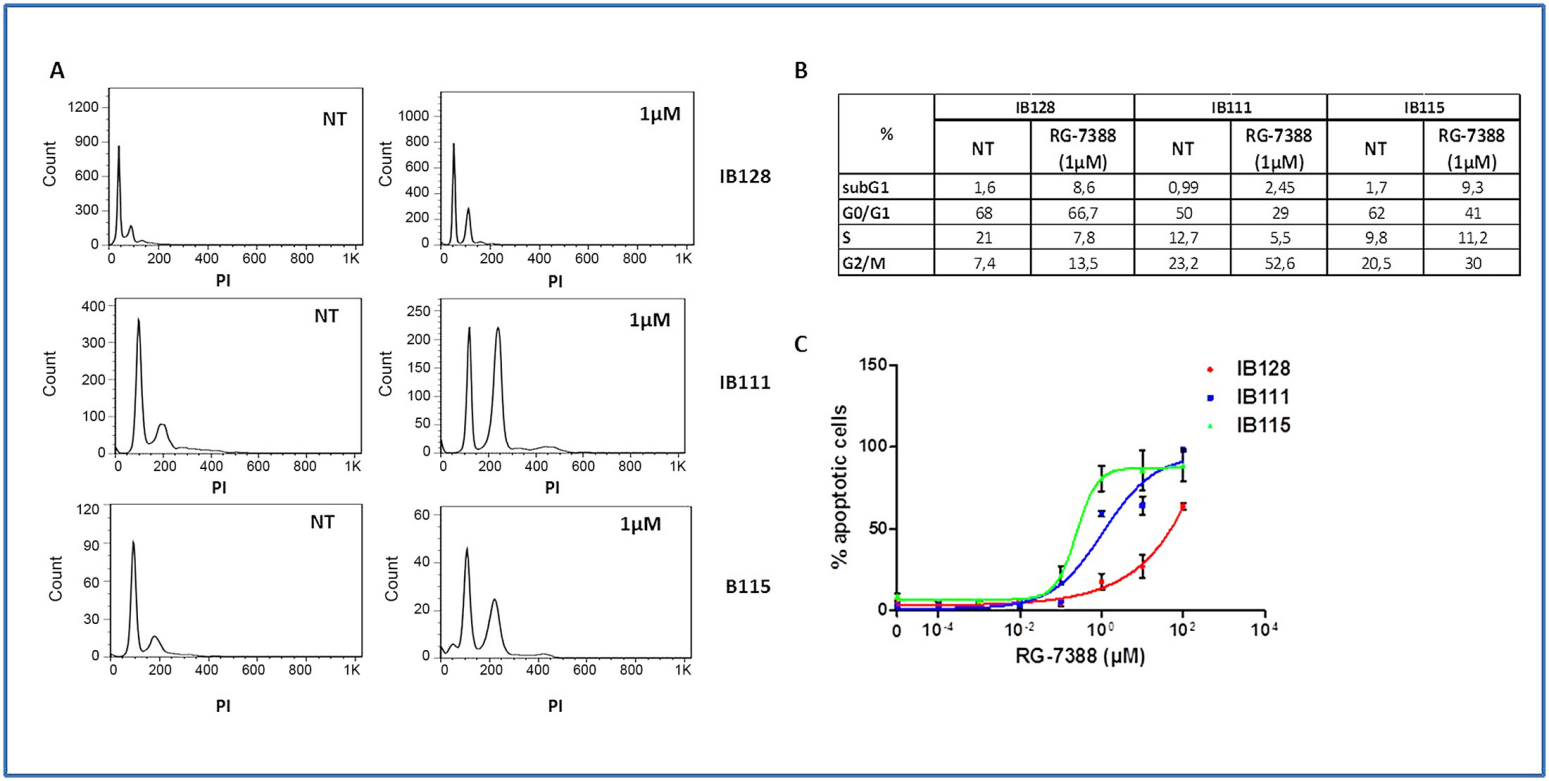
Effect of RG7388 on cell cycle progression and cell viability in human STS cell lines. (A) Cell cycle profile before and after treatment with 1μM of RG7388 analyzed by PI incorporation and flow cytometry in the IB111, IB115 and IB128 cell lines. (B) Cell-cycle distribution was calculated from the flow cytogram. (C) Effect of RG7388 on loss of potential mitochondrial membrane with TMRM fluorescent assay in the IB111, IB115 and IB128 cell lines.

One of the main cellular functions of activated TP53 is blocking cell cycle progression in the G1 and G2 phase. Treatment of exponentially proliferating STS cell lines with RG7388 for 48 hours led to a dose-dependent cell cycle block in G1 and G2/M phase and a depletion of the S phase compartment (Fig 2A and 2B). One of the other main functions of activated TP53 is induction of apoptosis. Exposure of exponentially proliferating STS cell lines to RG7388 RO5503781 for 72 hours led to the induction of apoptosis in a dose-dependent manner as revealed by an increase in the percentage of TMRM-staining cells (Fig 2C). The 3 STS cell lines that elicited the most significant apoptotic responses were IB111, IB115 and IB128.

### Repeated exposures of STS cell lines sensitive to RG7388 lead to the emergence of sub-cell lines strongly resistant to the RG7388 anti-tumor activity

**Fig 3:**
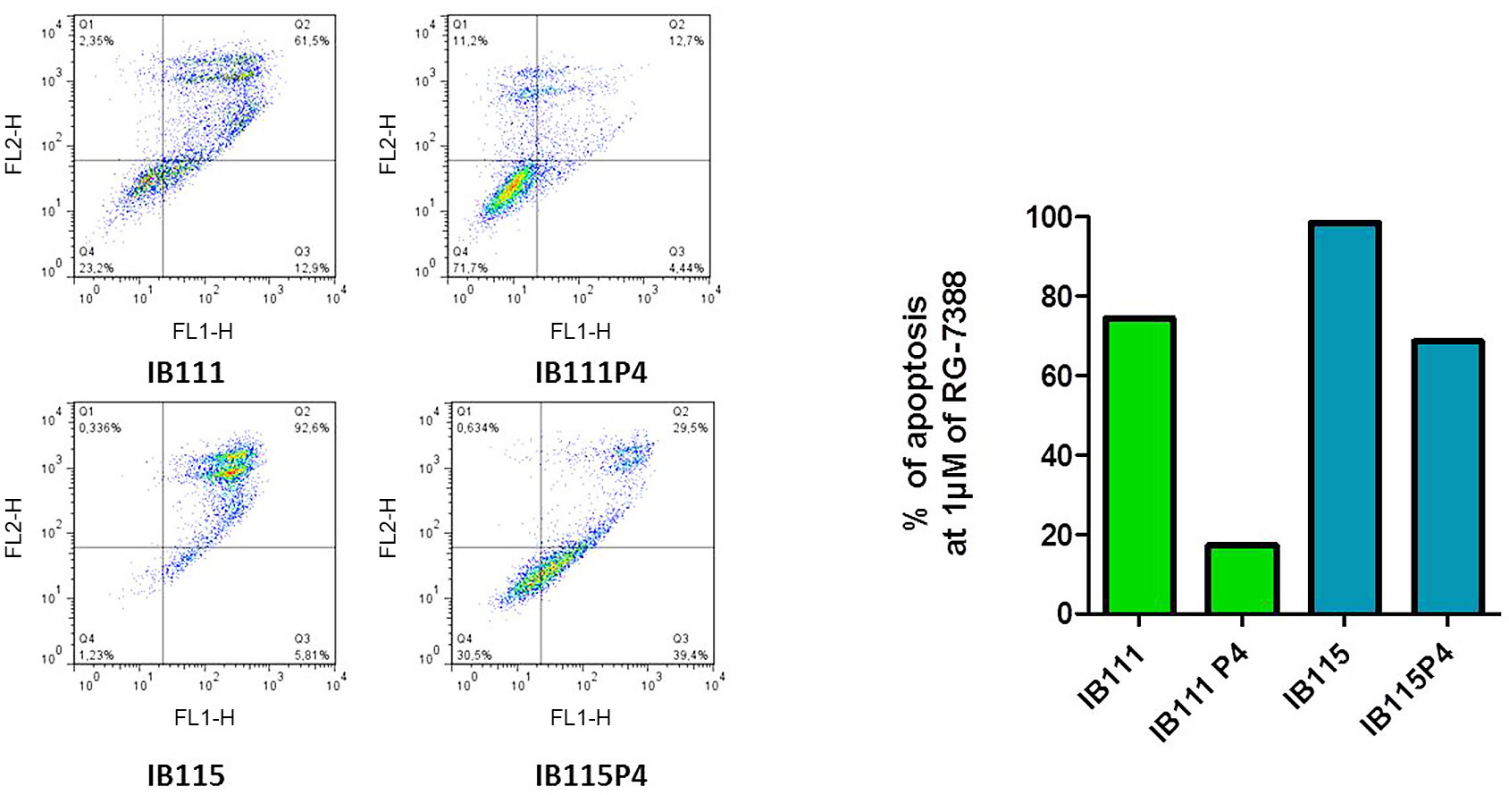
(A) Antiproliferative activity and impact on loss of potential mitochondrial membrane with TMRM fluorescent assay (cell viability) (B) of RG7388 in parental and secondary resistant IB111, IB115 and IB128 STS cell lines. (C) Apoptosis induction analysis using FITC annexin-V and propidium iodide assay. Sensitive (IB111 and IB115) and resistant cells (IB111P4 and IB115P4) were treated 72h by RG-7388 at 1μM.

Among the entire panel of STS cell lines tested, RG7388 displayed the highest cell growth inhibition effects in IB111, IB115 and IB128. In order to investigate mechanisms of secondary resistance to RG7388, IB111, IB115 and IB128 cells were treated with RG7388 for 3 days. The cells were then rinsed to remove RG7388, and the remaining cells were expanded in normal medium (minus RG7388). We repeated the process four times, and we obtained populations that survived 1–4 rounds of RG7388 treatment (P1 for one round—P4 for four rounds). We compared the extent to which IB111, IB115 and IB128 underwent apoptosis and cell cycle inhibition when treated for 3 days with RG7388. Results indicated that the selected populations became progressively more resistant to cell growth inhibition (Fig 3A) to apoptosis (Fig 3B) and cell cycle inhibition (data not shown). For instance, whereas 1 μM of RG7388 induced significant apoptosis in parental IB115 cells (80.7% apoptosis) after 3 days of treatment with RG7388, this effect was significantly reduced in IB115P2 (36.7% apoptosis) and IB111P4 (8.8% apoptosis) cells. Beside, we confirmed resistance to apoptosis at 1μM of RG7388 in resistant cell lines IB115P4 (69.4% apoptosis) and IB111P4 (28.4% apoptosis) versus parental cell lines IB115 (98.7% apoptosis) and IB111 (76.7% apoptosis) by FITC annexin-V and propidium iodide assay (Fig 3C).

### STS sub-lines with secondary resistance to RG7388 display sensitivity to cytotoxic drugs similar to the parental cell lines

In order to assess specificity of secondary resistance to RG7388, we compare the sensitivity profiles of IB111, IB115, IB128 and their RG7388-resistant counterparts IB111P4, IB115P4 and IB128P4 to doxorubicin and gemcitabine, two cytotoxic drugs commonly used to treat STS patients with advanced disease. Doxorubicin and gemcitabine significantly inhibited the proliferation with the same extent in the parental cell lines and the RG7388-resistant sublines (S1 Fig). This indicated that secondary resistance to RG7388 in IB111P4, IB115P4, IB128P4 did not result from a multidrug resistance phenotype and may involve specific mechanisms related to the mechanisms of action of RG7388.

### Deep sequencing identifies ploidy variations involved in secondary resistance of STS cells to RG7388

Several studies have shown correlations between gains and losses of chromosomes and cancer-specific drug resistance [27–29]. We wanted to investigate whether regional ploidy variations were associated with secondary resistance to RG7388 in STS cells. Results are summarized in Table 1 and S2 Fig. IB111 had the greater number of allelic imbalances, followed by IB115 and IB128. Some of them were shared by both the parental and secondary resistant cell lines. However, all the secondary resistant cell lines were characterized by at least one specific ploidy variation not found in the parental ones. For instance, the secondary resistant IB111 cell line was also characterized by an unbalanced tetraploidy of the 12q13.2-qter region in which MDM2 is located. The secondary resistant IB128 cell line was also characterized by an unbalanced pentaploidy of the short arm of chromosome 17 including the *TP53* gene.

**Table 1:** Ploidy variations between secondary resistant and parental soft-tissue sarcoma cell lines.

Genomic region	IB111		IB115		IB128	
	Sensitive	Resistant	Sensitive	Resistant	Sensitive	Resistant
chr1p36.33-p36.22	-	**tri-ploidy**	-	-	-	-
chr4q22.3-qTer	-	**tetra-ploidy**	-	-	-	-
chr5	-	**tetra-ploidy**	-	-	-	-
chr7q11.23-qTer	-	-	-	**tetra-ploidy**	-	-
chr8q	*tetra-ploidy*	**deca-ploidy**	-	-	-	-
chr10q22.2-qTer	-	**tetra-ploidy**	-	-	-	-
chr12q13.2-qTer	-	**tetra-ploidy**	-	-	-	-
chr17p	-	-	-	-	-	**penta-ploidy**
chr22q	*tri-ploidy*	-	-	-	-	-

*only sensitive clone*

**only resistant clone**

-: absence of allelic imbalance

### Secondary resistance of STS cells to RG7388 is characterized by clonal selection of cancer gene mutations

**Fig 4:**
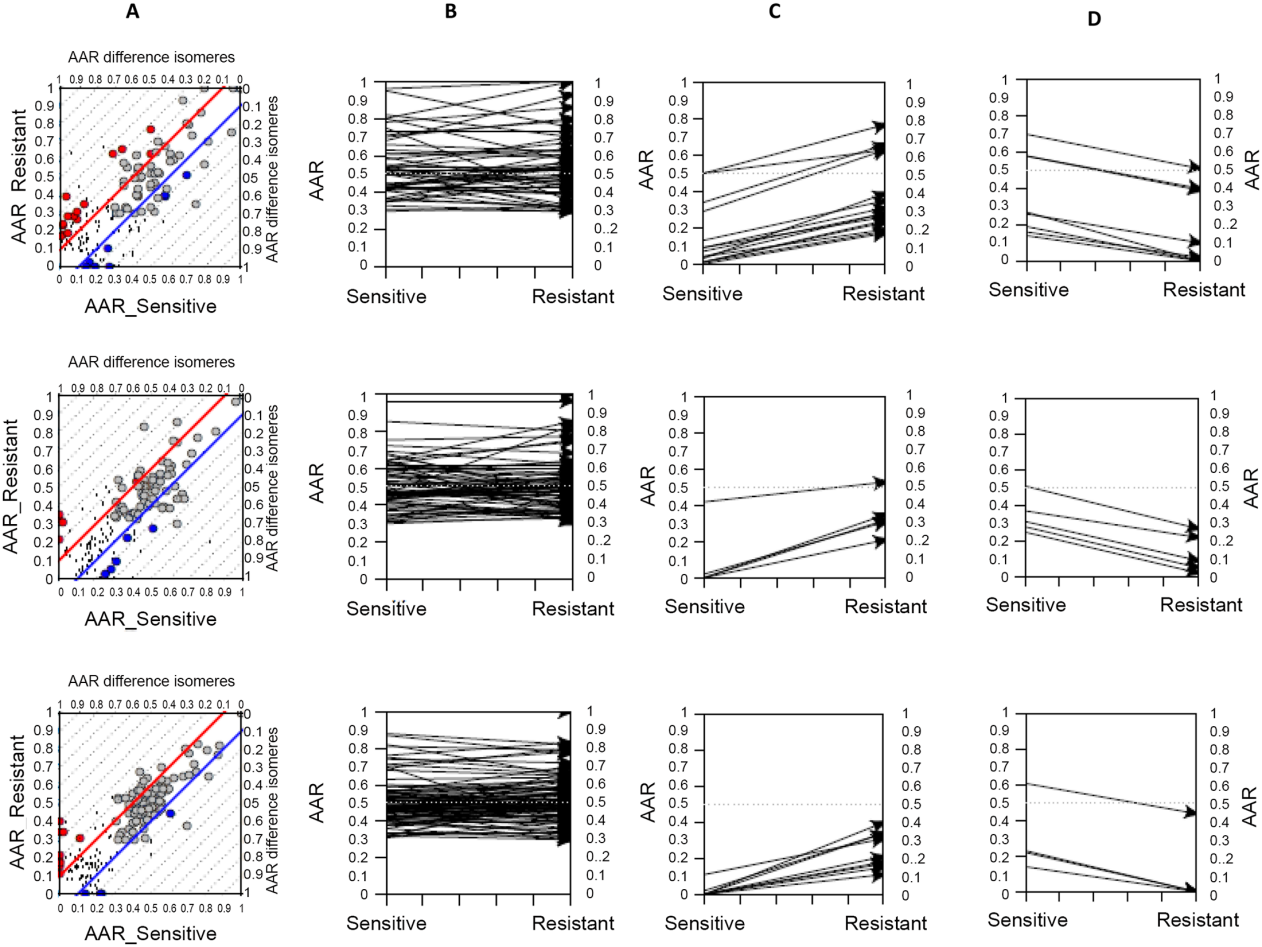
Variants in sensitive and secondary resistant STS cells. From top to bottom rows, plots of IB111, IB115 and IB128 variants. A. AAR scatters parental (X axis) and resistant (Y axis), red points: AAR higher in resistant, blue points: AAR lower in resistant, gray points: AAR unchanged (parental variants), dotted lines: AAR difference isomers (lines of same AAR difference). B. Parental variants, on Y axis the AAR in parental and resistant samples. C. variants whose AAR increases in resistant samples (15 in IB111, 5 in IB115, 9 in IB128) D. variants whose AAR decreases in resistant samples.

To identify changes in the mutation profiles of secondary resistant cells, we compared the abundance of somatic mutations found in the parental and secondary resistant cell lines. For each cell line, we examined a conservative list of mutations and used Fisher’s exact test to identify those specifically associated with secondary resistance (see material and methods). In all cell lines the majority of variants were common to the parental and resistant cells (Fig 4). The number of non-synonymous mutations with significant changes in normalized abundance between parental and resistant cell lines ranged from 10 to 23 for each case. These include mutations in well-known cancer genes, genes linked to drug resistance and drug metabolism, and genes not previously associated with carcinogenesis or therapy resistance (Table 2). The *TP53* gene was the only recurrent gene we identified as having mutations with significant changes in abundance between the secondary resistant and the parental cell lines. Indeed, we identified one TP53 mutation in the IB115 cell line (C275S) and three different TP53 mutations in the IB128 cell line (R248P, G199E, T125R). These mutations have all been previously reported in human tumor samples (http://cancer.sanger.ac.uk/wgs/gene/analysis?ln=TP53#dist) and are located in the highly- conserved DNA binding domain of TP53. All these positions have been validated with ultra-deep DNA resequencing of the TP53 coding region (data not shown). No TP53 mutations were identified in the IB111 cell line.

**Table 2:** Selected mutations whose mutant AF significantly increased following treatment with RG7388.

				Mutant AF	
CELL LINE	GENE	TRANSCRIPT	Entrez Gene Name	Sensible	Resistant
IB111	*HSPG2*	*NM_005529*:*exon42*:*c*.*A5239C*:*p*.*T1747P*	heparan sulfate proteoglycan 2	0.03	0.39
	*MACF1*	*NM_012090*:*exon91*:*c*.*A15886C*:*p*.*T5296P*	microtubule-actin crosslinking factor 1	0.01	0.23
	*P4HTM*	*NM_177938*:*exon6*:*c*.*C933G*:*p*.*S311R*	prolyl 4-hydroxylase, transmembrane (endoplasmic reticulum)	0.09	0.26
	*ECT2*	*NM_001258315*:*exon4*:*c*.*T245C*:*p*.*I82T*	epithelial cell transforming 2	0.01	0.18
	*HLA-A*	*NM_001242758*:*exon6*:*c*.*A1033T*:*p*.*T345S*	major histocompatibility complex, class I, A	0.3	0.95
	*HLA-C*	*NM_002117*:*exon2*:*c*.*G97T*:*p*.*D33Y*	major histocompatibility complex, class I, C	0.21	0.88
	*SUPV3L1*	*NM_003171*:*exon5*:*c*.*G680C*:*p*.*G227A*	suppressor of var1, 3-like 1 (S. cerevisiae)	0.29	0.63
	*PHLDB1*	*NM_001144759*:*exon17*:*c*.*C3418T*:*p*.*R1140C*	pleckstrin homology-like domain, family B, member 1	0.34	0.66
	*ATP5B*	*NM_001686*:*exon8*:*c*.*A1123G*:*p*.*T375A*	ATP synthase, H+ transporting, mitochondrial F1 complex, beta polypeptide	0.5	0.63
	*GPN3*	*NM_016301*:*exon5*:*c*.*A512C*:*p*.*N171T*	GPN-loop GTPase 3	0.5	0.77
	*POLG*	*NM_001126131*:*exon14*:*c*.*G2323A*:*p*.*E775K*	polymerase (DNA directed), gamma	0.01	0.24
	*PKD1*	*NM_001009944*:*exon28*:*c*.*T9583C*:*p*.*W3195R*	polycystic kidney disease 1 (autosomal dominant)	0.07	0.19
	*ZNF205*	*NM_001042428*:*exon7*:*c*.*A1204C*:*p*.*T402P*	zinc finger protein 205	0	0.17
	*PSMB10*	*NM_002801*:*exon3*:*c*.*C172G*:*p*.*R58G*	proteasome (prosome, macropain) subunit, beta type, 10	0.07	0.28
	*SF3B3*	*NM_012426*:*exon3*:*c*.*C382G*:*p*.*R128G*	splicing factor 3b, subunit 3, 130kDa	0.09	0.31
	*SLC38A10*	*NM_001037984*:*exon4*:*c*.*G349T*:*p*.*G117W*	solute carrier family 38, member 10	0.04	0.19
	*NR2F6*	*NM_005234*:*exon1*:*c*.*A271C*:*p*.*T91P*	nuclear receptor subfamily 2, group F, member 6	0.13	0.35
	*AP1B1*	*NM_001127*:*exon5*:*c*.*C369G*:*p*.*C123W*	adaptor-related protein complex 1, beta 1 subunit	0.04	0.28
IB115	*CKAP2L*	*NM_152515*:*exon3*:*c*.*C119A*:*p*.*S40Y*	cytoskeleton associated protein 2-like	0	0.32
	*PDLIM4*	*NM_001131027*:*exon6*:*c*.*C674T*:*p*.*A225V*	PDZ and LIM domain 4	0.42	0.53
	*FAM45A*	*NM_207009*:*exon8*:*c*.*G810A*:*p*.*M270I*	family with sequence similarity 45, member A	0	0.21
	*TP53*	*NM_001276698*:*exon4*:*c*.*G347C*:*p*.*C116S*	tumor protein p53	0	0.35
	*EMC10*	*NM_175063*:*exon5*:*c*.*G529T*:*p*.*D177Y*	ER membrane protein complex subunit 10	0.02	0.31
IB128	*UBE4B*	*NM_001105562*:*exon4*:*c*.*G367C*:*p*.*D123H*	ubiquitination factor E4B	0	0.18
	*NRAS*	*NM_002524*:*exon3*:*c*.*A182G*:*p*.*Q61R*	neuroblastoma RAS viral (v-ras) oncogene homolog	0	0.15
	*ENAH*	*NM_001008493*:*exon6*:*c*.*C836T*:*p*.*S279F*	enabled homolog (Drosophila)	0	0.11
	*SYNJ2*	*NM_003898*:*exon4*:*c*.*A487G*:*p*.*N163D*	synaptojanin 2	0.11	0.31
	*HPD*	*NM_001171993*:*exon16*:*c*.*G967A*:*p*.*G323S*	4-hydroxyphenylpyruvate dioxygenase	0	0.34
	*TP53*	*NM_001276698*:*exon3*:*c*.*G266C*:*p*.*R89P*	tumor protein p53	0	0.21
	*TP53*	*NM_001276698*:*exon2*:*c*.*G119A*:*p*.*G40E*	tumor protein p53	0	0.17
	*TP53*	*NM_001126112*:*exon4*:*c*.*C374G*:*p*.*T125R*	tumor protein p53	0	0.4
	*C17orf89*	*NM_001086521*:*exon1*:*c*.*C68G*:*p*.*A23G*	chromosome 17 open reading frame 89	0.02	0.34

### Gene expression analysis identifies defects in apoptosome activity and autophagy induction as a mechanism of secondary resistance to RG7388

**Fig 5:**
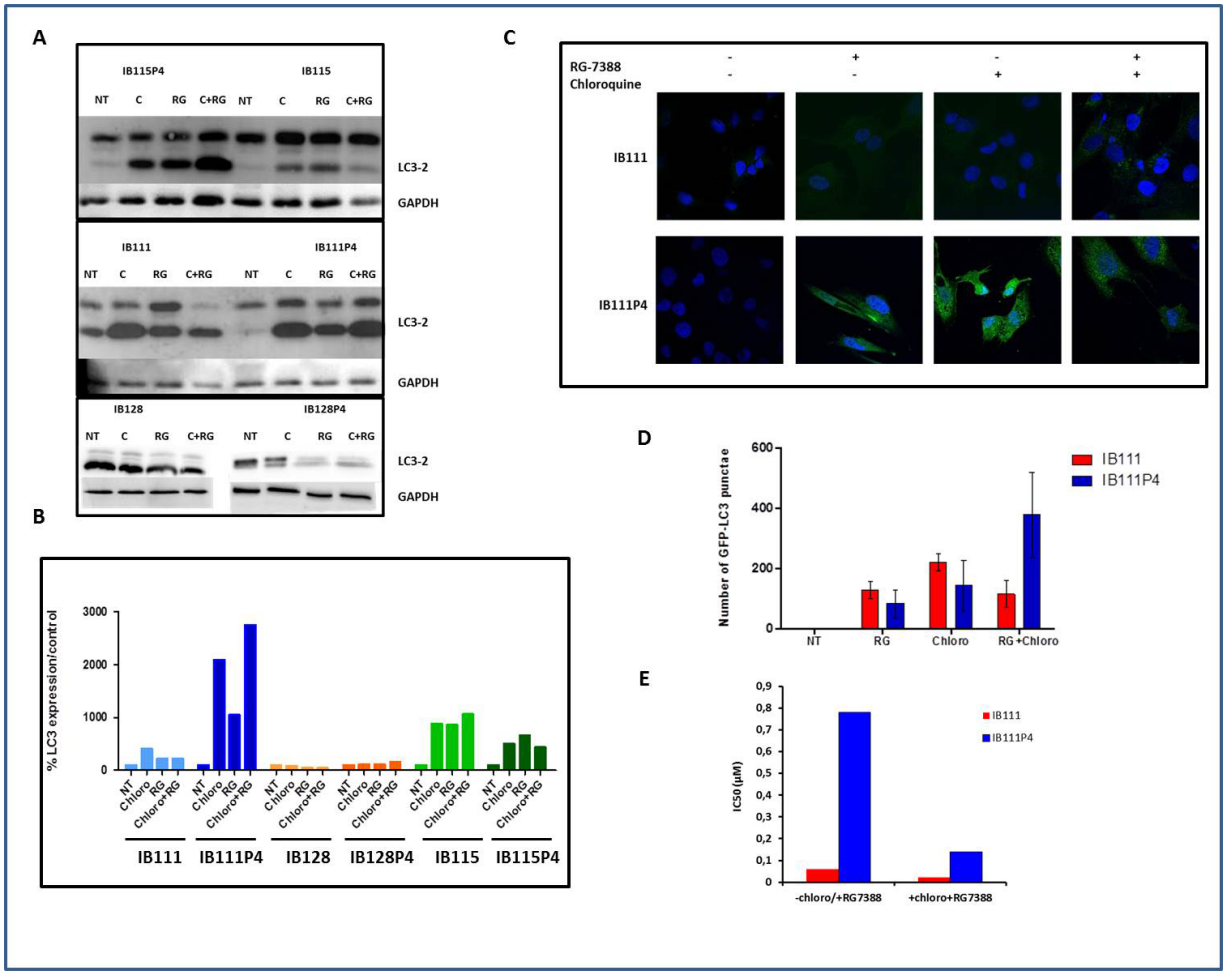
Secondary resistance to RG7388 is associated with autophagy induction. (A) parental and secondary resistant cells were incubated with 20μM of chloroquine for 6h, or RG7388 alone for 72h or RG7388 (over 72h) and chloroquine for 6h before protein extraction, and immunoblot. (B) Densitometry of the immunoblot, the graph represents the percentage of LC3-II /GAPDH relative to the untreated control (C) IB111 and IB111P4 were treated with 1μM and 10 μM of RG-7388 respectively with or without chloroquine and fixed for immuno-staining with LC3 antibody (D) Quantification of autophagy induction (number of GFP-LC3 punctuae) in IB111 and IB111P4 treated with RG7388, chloroquine and RG7388+chloroquine (E) IC50 fold-change related to antiproliferative activity of RG7388+chloroquine versus RG7388 alone in secondary resistant IB111, and sensitive cells IB111.

We also analyzed the sequencing data to identify differences in gene expression between secondary resistant and parental cell lines. We found 196, 105 and 370 genes with 2.5X or more fold changes (adjusted p-value < 0.01) between the parental and secondary resistant cell lines in IB111, IB115 and IB128 respectively (S2 Table). We then applied these genes to Ingenuity Pathway Analysis software (IPA^®^,QIAGEN Redwood City, www.qiagen.com/ingenuity). The results showed that these genes were mainly enriched in proliferative, growth and movement networks (Table 3). We focus particularly on the genes differentially expressed between the resistant and parental IB111 cells in order to identify some genetic and transcriptomic alterations that may compensate for the lack of TP53 mutations observed in the IB111 resistant cells. Strikingly, in the resistant IB111 cells, we found a significant alteration of genes involved in the regulation of apoptosis and more particularly, a down-regulation of pro-apoptotic activators such as *BMF*, *BIM* and *PUMA* (S3 Fig). Among them, the most strongly down-regulated was *BMF* with a fold change of 17. BMF also has a role as a regulator of autophagy. Since autophagy has been associated with resistance to radiation, chemotherapy, and targeted agents [30–39], we decided to investigate whether secondary resistance to RG7388 was associated with autophagy induction in STS cell lines. When autophagy is not activated, LC3 is localized homogeneously in the cytoplasm, while upon initiation of autophagy, it associates with the membrane of autophagosomes. Since an increase in LC3-II levels or GFP-LC3 vesicles can occur not only due to increased autophagosome synthesis but also due to impaired autophagosome-lysosome fusion, we assessed LC3-II levels also in the presence of chloroquine, a blocker of LC3-II degradation. Analysis of LC3-II levels by western blotting and by fluorescence microscopy allowed us to detect autophagy induction in the resistant IB111 cells but not in the IB115 and the IB128 resistant cells (Fig 5A and 5B). We therefore wondered whether blocking the induction of autophagy with chloroquine could restore sensitivity to RG7388 in IB111 resistant cells. The RG7388 IC50's were significantly reduced (divided by 6) in the secondary resistant IB111 cells when they were treated with chloroquine whereas RG7388 IC50 was almost unchanged in parental IB111 cells treated with chloroquine in the same conditions. (Fig 5C).

**Table 3:** Differential gene expression and pathways enriched in STS cells secondary resistant to RG7388.

ID	Molecules in Network	Score	mol.	Top Diseases and Functions
IB111				
1	Akt, BCR (complex), **BMF**, **CCL26**, **CCNG2**, **DCN**, **DPP4**, **EGR1**, ERK, ERK1/2, **FBXO32**, Focal adhesion kinase, **GBP2**, **GLIPR2**, Hdac, **IGFBP3**, **IL6**, **IL20RB**, **ITGAV**, **JUNB**, LUM, Mek, **NOTCH3**, P38 MAPK, **PCDH18**, PDGF BB, **PDGFRB**, PI3K (complex), PI3K (family), **PTGES**, **RRAD**, **RUNX2**, *S1PR1*, **TNS1**, **TSC22D1**	37	24	Cellular Growth and Proliferation, Tissue Development, Cellular Development
2	ADCY, *ADORA2A*, **AMIGO2**, Ap1, **CCL2**, CD3, **CD9**, **CD74**, Cg, collagen, **CTSK**, CTSS, *CXCL1*, **ERAP1**, Fibrinogen, *G0S2*, **GATA6**, **HTRA1**, IL12 (complex), *INHBB*, Jnk, *KRT17*, LDL, Mapk, *NFATC1*, NFkB (complex), NOV, Pkc(s), *PLAT*, **PSMB9**, **RARRES3**, **S100A4**, **TFPI2**, **TIMP3**, **WISP1**	35	23	Cellular Movement, Skeletal and Muscular System Development and Function, Connective Tissue Disorders
3	ADA, ATG7, **BAMBI**, **CCL26**, CITED2, COPS5, **FAM46A**, **FHL1**, GADD45A, GLI1, **GPNMB**, **GPX3**, **GREM2**, **HAPLN3**, **HHIPL2**, IL13, KIAA1524, **LUM**, MAPK8, MXD1, PAF1, **PARP14**, **PCOLCE2**, PDLIM2, **PDZRN3**, PHLDA1, *PSTPIP2*, PTP4A1, **S100A2**, **SLC12A8**, SMARCA4, TGFB2, **TNS1**, TP53, **YPEL3**	24	18	Cell Morphology, Cellular Assembly and Organization, Developmental Disorder
4	**ARRDC3**, B4GALT5, BUB1, BUB1B, COPS5, **DBP**, **DDIT4**, ERBB2, **ERRFI1**, *ETV4*, **ING4**, ITGA2, ITGB4, *KRT81*, mir-29, mir-145, MIXL1, MSH6, **NREP**, PADI4, **PDE4B**, PDK1, POLE2, PRDM5, **PTN**, **S100A2**, **SCG5**, SLC2A12, SP1, TGFB2, **TMSB15A**, TOP2A, TP53, **UCHL1**, **WNT10B**	19	15	Cancer, Organismal Injury and Abnormalities, Reproductive System Disease
5	**ARHGAP24**, **CDA**, CDH1, CDK5R1, CLDN2, Collagen type III, **CTHRC1**, CTNNB1, ECM1, **ENPEP**, ERG, **FZD6**, HNF1A, **HTRA1**, *INHBB*, **ITGA11**, ITGB1, **LBH**, MAGI1, MCAM, **MSX2**, **PBX1**, PRDX2, SERPINA3, **SLC39A10**, *SLIT2*, SNAI1, SUZ12, SYVN1, TCF, THBD, **TSC22D1**, **TUBA4A**, UGT2B17, WNT2	19	15	Embryonic Development, Hair and Skin Development and Function, Organ Development
6	26s Proteasome, AR, AREG, AZGP1, **BTN3A3**, COL17A1, **CRABP2**, **CTSO**, EGFR, **GREM1**, HBEGF, **HECW2**, HSPA5, HTT, IgG, ISG15, *KRT17*, *KRT34*, **LAMA4**, MAPK1, NKX3-1, OASL, **PITX2**, PLD1, **PSMB8**, **PXK**, RARA, SFPQ, **TCF7**, THBD, TMEM158, TREM1, *UCP2*, VDR, WNT5A	15	13	Organismal Development, Developmental Disorder, Cellular Growth and Proliferation
7	ADCY9, ANK1, **APOL6**, **AQP3**, BECN1, **BTN3A1**, CENPC, **CYP1B1**, **DHRS3**, ELL2, ERN1, FSH, **GBP2**, GK, **HDAC5**, Histone h3, IFNG, ING2, INHA, Lh, **LPCAT2**, *MT1X*, *MT2A*, **NUPR1**, P4HA2, PTP4A1, RAPGEF3, RASAL2, RNA polymerase II, **SOCS2**, SPATS2L, STK17A, TLN1, UPP1, **WNT7B**	15	13	Dermatological Diseases and Conditions, Cell Morphology, Cellular Function and Maintenance
8	ADORA2B, **AMIGO2**, **APOL1**, **C1QTNF1**, **CASP1**, **CD248**, **CEMIP**, CORO1C, CSF2, CXCL8, *DENND2A*, EZH2, GADD45A, **GLIPR1**, HIC1, IL18RAP, Interferon alpha, *KRT81*, LGALS3BP, **LPPR4**, MAL, MBD3, NLRC4, NLRP3, NR3C1, PLA2G4A, PYCARD, RIPK2, *SCD*, SERPINB9, **SNTB1**, TLR1, TLR5, TLR6, **TRIM16L**	15	13	Cellular Function and Maintenance, Hematological System Development and Function, Gastrointestinal Disease
9	ABCG2, ANXA2, **C19orf66**, **CPE**, CXCL3, CXCL11, DUSP4, E2F1, E2F4, ESR1, **FAM198B**, **FOXG1**, GINS1, **HIST1H2AC**, **HIST2H2BE**, **HLA-DMA**, HLTF, **IFI35**, IFNL1, IL27, JUN, LRIG1, **LRIG3**, MCM10, **MFAP4**, NCOA3, NEUROG1, OASL, POLA2, **PSMB9**, PTX3, **SAMD11**, **SLC38A2**, SMC4, TNF	15	13	Cancer, Endocrine System Disorders, Organismal Injury and Abnormalities
10	**ABRACL**, **BBC3**, BIK, BRD4, **C9orf3**, **CD24**, CD79A, COL5A1, **COL5A3**, CTCF, DICER1, estrogen receptor, **FKBP11**, FLI1, GADD45A, **ID2**, *KRTAP2-3/KRTAP2-4*, miR-145-5p (and other miRNAs w/seed UCCAGUU), **MSC**, MYC, NFYA, NFYB, NFYC, NTRK2, P-TEFb, PADI4, **PBXIP1**, PDX1, POU2F2, RARRES1, SLC4A4, **SLIT3**, **SSBP2**, TCF3, TIMP4	14	12	Cellular Development, Hematological System Development and Function, Hematopoiesis
IB115				
1	**A4GALT**, **APOD**, **ARHGDIB**, **CD74**, **CTGF**, ERK, *FBLN2*, *GBP1*, **HTATIP2**, **HTRA1**, **IFI6**, **IFI27**, IFN Beta, Interferon alpha, **ITGB2**, *LCN2*, LDL, *LPIN2*, lymphotoxin-alpha1-beta2, Mapk, **MMP1**, *NCF2*, NFkB (complex), PI3K (complex), **PRKCB**, PYCARD, **RAC2**, Ras, SAA, **STAT6**, TCR, **TIMP3**, **TRIB2**, **USP18**, Vegf	42	22	Ophthalmic Disease, Cell Death and Survival, Dermatological Diseases and Conditions
2	Akt, Ap1, **ASGR1**, *CD55*, Cg, *COL15A1*, **CRABP2**, *CYP1A1*, **DBNDD1**, **DCN**, estrogen receptor, Focal adhesion kinase, FSH, **GATA6**, Gm-csf, GPRC5B, Histone h3, Hsp70, IgG, **IL6**, *IL1B*, ITGB4, Jnk, Lh, MAP2K1/2, P38 MAPK, PDGF BB, **PDGFA**, *PTPRN*, **RGS16**, **RNASE1**, **RRAD**, **SERPINH1**, *TGFA*, TMEM158	34	18	Cellular Development, Cellular Growth and Proliferation, Organ Morphology
3	ALB, BGN, *C1S*, **COL14A1**, **COL1A2**, FBN1, FLI1, FOS, GLI1, GLI2, **GNAO1**, GPER1, **GREM1**, HAS1, *INHBE*, **ITGB2**, MAPK1, MAPK13, **MEST**, MMP10, PTH, *RBP7*, *RHOU*, RPS6KA1, RXRA, SBDS, *SFRP1*, SNAI2, *TGFA*, TGFB1, THBD, **TMEM100**, TMEM158, TREM1, WNT5A	23	14	Cellular Movement, Organismal Development, Skeletal and Muscular System Development and Function
4	*APBA2*, APP, BCL2, Beta Secretase, **BMP8B**, C3, *CD55*, CEBPA, CHI3L1, CLSTN1, **CRIP1**, **CRIP2**, **CTSL**, **CXCL14**, ESR1, FOSL2, GAB2, **GJB2**, HAMP, HSPA8, IL32, KDM6A, KMT2D, KRT14, *LTBP1*, **NES**, **NFATC4**, PAXIP1, PINK1, PTX3, *SERPINB1*, SMAD6, SNAI2, *TMOD1*, TNFAIP6	19	13	Cellular Function and Maintenance, Cellular Movement, Cellular Growth and Proliferation
5	CCL22, CD3, CYP19A1, **EDN2**, **EPB41L3**, ERK1/2, FLOT1, **FLOT2**, FPR2, FYB, FYN, **HOMER2**, IL9, IL36G, LITAF, MAL, **MVB12B**, NEDD4L, **NPPB**, NPR3, NR3C1, PDLIM2, PTGER4, PTK2B, RASA1, **SKAP1**, **SMOC1**, **SPOCD1**, THBD, THY1, TSG101, **TXNIP**, VPS28, VPS37B, YWHAG	13	10	Cellular Movement, Hematological System Development and Function, Immune Cell Trafficking
IB128				
1	*ALDH1A3*, *ANXA1*, Ap1, Cg, *CLEC11A*, Collagen type II, *CXCL2*, *CXCL3*, **CXCL16**, *F3*, *FPR1*, *G0S2*, Ikb, *IL11*, *IL1A*, *ITGA2*, *LCN2*, lymphotoxin-alpha1-beta2, **MMP1**, NFkB (complex), *NLRP3*, *OTUB2*, *PHLDA1*, PLC, *PODXL*, *PPAP2B*, *RAB31*, *RGS4*, *RGS20*, SAA, **SLC7A5**, *STC1*, *TFPI2*, *TM4SF1*, *TMEM158*	37	27	Cellular Movement, Hematological System Development and Function, Immune Cell Trafficking
2	*ACSL5*, *ADM*, Akt, *ANKRD1*, **ARHGDIB**, *BCL2A1*, BCR (complex), CD3, **EFNA3**, *FKBP11*, *FST*, Gm-csf, *GPRC5A*, Iga, IgG, *IL24*, IL12 (complex), **IL21R**, *KRT15*, *LPXN*, *MGLL*, **MMP9**, N-cor, Notch, **NOTCH3**, **PDE4A**, Pkc(s), *PMAIP1*, *PPP1R15A*, *PTGS2*, **RND2**, *SERPINB7*, *SLAMF7*, *SLC22A17*, **TAGLN**	32	25	Cancer, Gastrointestinal Disease, Organismal Injury and Abnormalities
3	ACKR3, *AKR1B10*, *AKR1C1/AKR1C2*, *CCL26*, **COL3A1**, Creb, *CRMP1*, Ctbp, **CXCL12**, *DDX58*, *DKK1*, ERK1/2, *F2R*, **FGFR4**, Focal adhesion kinase, G-protein beta, *GPR56*, *GRK5*, *HBEGF*, *HMMR*, *IFIT1*, IFN Beta, Igm, IL1, *IL6ST*, *IL7R*, *ITGA6*, **NTN1**, P38 MAPK, PI3K (family), Ras homolog, *SFRP1*, *SPRY2*, **SULF1**, **SULF2**	30	24	Cellular Movement, Digestive System Development and Function, Organ Morphology
4	*AKAP12*, **BGN**, *BMP7*, *CARD16*, *CASP1*, *CLDN4*, **CNTN1**, Cpla2, *CXCL8*, *DUSP10*, *FBLN2*, *GDF15*, Gsk3, Hdac, Histone h4, *HOXA9*, **HOXB9**, **HTATIP2**, IL32, *IL1B*, Jnk, KCNMA1, LDL, Mapk, **MGAT3**, *NOV*, *PREX1*, *PRKACB*, Rac, Ras, **SERPINA1**, *SPP1*, *STMN2*, TMSB4, Vegf	30	23	Cellular Movement, Cancer, Cellular Growth and Proliferation
5	26s Proteasome, **ACTA2**, **ACTG2**, *ANGPTL4*, *APOBEC3G*, *APOL3*, *C12orf29*, *CD55*, CLTCL1, *DHRS3*, **DUSP9**, estrogen receptor, FSH, Growth hormone, Hsp70, Hsp90, *IFI44*, *IFIH1*, *IFIT2*, *IFIT3*, Immunoglobulin, Interferon alpha, *ISG20*, *LGALS9*, Lh, *PRKAG2*, *RARRES3*, **RBP1**, **RGS16**, STAT5a/b, TCR, **TGFB3**, *TGM2*, *TNFRSF11B*, UBD	30	24	Antimicrobial Response, Inflammatory Response, Infectious Disease
6	*ABCG2*, *ADORA2B*, *ADRB2*, **ARRB1**, *CAMK2N1*, *CCL5*, Cdk, **CNN1**, Collagen type I, *CSF2*, *DUSP4*, *DUSP6*, EGR2, Eotaxin, ERK, Fcer1, Fcgr2, *HAS3*, HLA-DQ, Hsp27, *IGF2BP3*, *IL6*, *LIF*, Mek, *MMD*, Nr1h, PDGF BB, **PDGFRB**, PI3K (complex), *RND3*, SCAVENGER receptor CLASS A, *SERPINB2*, *SGK1*, *TGFA*, Tlr	25	21	Cell Death and Survival, Cellular Development, Cellular Growth and Proliferation
7	**AARS**, **ATP2A3**, BCL6, CAV1, **CBS/LOC102724560**, **COL1A1**, **CTH**, CYP51A1, *DIRAS3*, GNAI2, HADHB, **HAPLN1**, HGF, INSR, *ITGA2*, *KRR1*, *LIF*, *MITF*, NOS3, **PCK2**, *PDCD1LG2*, PROCR, *RGS17*, SCARB1, SF1, SOX6, *SOX9*, SP1, **SREBF1**, STAT3, T, *TFPI*, *TNFRSF14*, TWIST1, ZBTB7B	20	18	Cardiovascular System Development and Function, Organismal Development, Cellular Growth and Proliferation
8	**ACKR3**, AR, *ARMCX2*, *C15orf48*, *CEND1*, COL11A1, DICER1, *DKK1*, EED, **EFHD1**, EGFR, ESR2, EZH2, FOXA1, *GBP3*, *GDF15*, *HBEGF*, HDAC2, HOXB5, ITCH, KCNA1, *KRTAP2-3/KRTAP2-4*, ***LAPTM5***, *LCN2*, MUC4, MYT1, *NCOA7*, RCOR1, RORA, **SEMA3F**, **SERPINA1**, *SFR1*, SUZ12, TCF7L2, *TNFRSF21*	20	18	Cellular Movement, Cellular Development, Cellular Growth and Proliferation
9	*AKR1C3*, APP, *BIK*, BSG, **CHAC1**, *COL13A1*, **COL16A1**, **COL5A1**, COL5A2, **COL5A3**, DPP4, EHD1, estrogen receptor, *FGF13*, FOS, HEXA, *HEXB*, *INHBE*, **ITGA11**, ITGB1, *KRT15*, *LY6K*, **MMP1**, MMP8, *NT5E*, RB1, SBDS, SCAVENGER receptor CLASS A, *SERPINB2*, *SNAPC1*, TNF, TNFAIP2, **TNK1**, UGCG, VEGFC	20	18	Connective Tissue Disorders, Lipid Metabolism, Molecular Transport
10	*BHLHE41*, CBX7, CCNT1, **CEMIP**, *CLDN1*, Cpla2, *DNER*, ENO2, FN1, GJA1, *GLIPR1*, HIF1A, HK2, HLX, HNRNPA2B1, *LETM2*, **MDFI**, mir-145, miR-145-5p (and other miRNAs w/seed UCCAGUU), *MITF*, MUC1, MYC, *PAPPA*, PDK1, *PITPNC1*, PPARGC1A, PRDM5, **PYCR1**, *RASSF8*, *SLC1A1*, *SOX9*, **STK32C**, *SYT1*, *UBASH3B*, WISP2	18	17	Cellular Movement, Cancer, Cellular Development
11	**ARG2**, **ASS1**, *ATF3*, C3, CAV1, CCL20, **CEMIP**, CXCL1, DICER1, *DYNLT3*, ELAVL1, ENPP2, EPAS1, F2RL1, *F2RL2*, **HEPH**, IL13, *IL1B*, **MFAP4**, *MYEOV*, NEUROG1, NOS2, PDLIM2, *PHLDA1*, PPARG, PRKCD, *SCG2*, *SDPR*, **SERPINF1**, SLC40A1, **SLC7A7**, *SMOC1*, *SPOCK1*, STAT1, THBS1	18	17	Cardiovascular System Development and Function, Organismal Development, Cell Death and Survival
12	**ASNS**, ATF2, *ATF3*, ATF4, *CD68*, CDC25C, **CNIH2**, **COL1A2**, CXCR5, *CYSTM1*, DDIT3, DNMT1, **DNMT3B**, GNE, HK2, JUND, *KLF4*, KLF6, MAP2K1, MAP2K1/2, NDRG1, **NUPR1**, ODC1, *PHLDA1*, **PODNL1**, PPP2R2A, **PSAT1**, **PSPH**, RAB39B, *RASGRF2*, RFX5, SAFB, *SAT1*, TRIB3, **UNC5B**	16	16	Cancer, Cell Morphology, Cellular Function and Maintenance
13	**ACTA2**, BTG2, BTRC, *C12orf5*, CASP7, *CCNE2*, CSE1L, DDB2, **DDIT4**, *DHRS2*, DLG1, DNMT1, FOSL1, *GGT1*, **GPR124**, HCAR2, *HMGA2*, **IL27RA**, JAK2, let-7, mir-23, **MMP9**, **NDUFA4L2**, PARP1, PEG10, RAF1, *RDH10*, RPL5, RPL11, *RRM2B*, S100A4, *SLC37A2*, SPHK2, TEP1, TP53	13	14	Cell Cycle, Cancer, Organismal Injury and Abnormalities
14	ACO2, AIM2, BMF, **CA9**, CAT, CHD4, CNOT7, **CSDC2**, *CTSS*, *CYP1A1*, CYP7A1, CYP8B1, *CYTH4*, DIO1, *HERC6*, Histone h3, IFNG, *IL1B*, MHC Class II (complex), **MMP11**, MRC1, *NCEH1*, OGG1, *OSGIN1*, PCSK1, PCSK2, PLA2G5, RNA polymerase II, *RTP4*, **SERPINH1**, SHH, *SLC14A1*, SOCS2, Sod, SUV39H1	12	13	Endocrine System Development and Function, Small Molecule Biochemistry, Cell-To-Cell Signaling and Interaction
15	*ABLIM3*, ACTG1, CCND1, CDH1, CEACAM1, *CLDN1*, CLDN7, *CLEC2B*, CTNNB1, *CXCL8*, EPCAM, ERN1, *EVI2A*, F5, FERMT2, *GLRX*, GNE, **GPT2**, **JDP2**, LGALS3, MGEA5, MMP2, MT1A, *NSA2*, PIAS1, *PLA2G16*, *RAB27B*, RELA, SATB1, *SPTLC3*, ST14, TFAP2A, TP53, TUBA4A	11	12	Cardiovascular Disease, Cardiovascular System Development and Function, Cell Morphology
16	*AGPAT9*, **CAMKK1**, CEBPA, CEBPD, CRTC1, CTTNBP2, CTTNBP2NL, *CYP1A1*, EIF4EBP1, *FGFR1OP2*, *GNG11*, HAMP, **KIF26B**, KLF16, LIG4, MOB4, NQO1, **NRTN**, PDCD10, **PER1**, RASAL2, SIKE1, STK24, STK25, *STRIP2*, STRN3, STRN4, *TBXAS1*, TERT, TRAF3IP3, **VLDLR**, *XRCC4*, XRCC5, XRCC6, YWHAG	11	12	Cellular Response to Therapeutics, Cell Morphology, Cellular Assembly and Organization

## Discussion

The new paradigm of targeted therapies has dramatically impacted oncology practice with the discovery and development of 'personalized' anti-cancer drug medicines that produce remarkable clinical responses in a subset of patients with advanced disease. While there is rapidly growing enthusiasm for this new paradigm, there is also increasing realization that such targeted therapies suffer from the same major limitation as traditional chemotherapy—clinical benefits are of limited duration due to secondary drug resistance.

Establishing specific molecular mechanisms of resistance to classical cytotoxic drugs has been challenging due to the nonspecific nature of their antitumor mechanisms of action. On the other hand, identification of mechanisms of acquired resistance to targeted therapies is crucial since the discovery of such mechanisms can prompt the development of strategies specifically designed to overcome them. The design of new drugs to treat lung cancer patients who become resistant to EGFR-directed therapy due to the emergence of the T790M secondary mutations represents a perfect example of the importance of research efforts in the field of secondary resistance [40].

In Phase I studies, RG7388 has been associated with disease control in a significant subset of sarcoma patients. However all of them had disease progression within a median of 6 months after treatment onset suggesting the occurrence of mechanisms of secondary resistance involved in tumor progression [14].

We report here the first comprehensive analysis of genetic mechanisms involved in secondary resistance to nutlin using next-generation sequencing of isogenic pairs of nutlin-sensitive and nutlin-resistant STS cell lines. The aberrations involved in secondary resistance we described here can be considered specific to RG7388 given that no cross-resistance was noted with other drugs commonly used for the management of STS patients such as doxorubicin or gemcitabine.

By performing deep sequencing of secondary resistant and parental sensitive cell lines, we showed here that secondary resistance to nutlin was associated with several mutations whose mutant allele fraction significantly changed between parental and secondary resistant cells. Among them, we identified TP53 mutations in the secondary resistant cells that were not present in the parental counterpart. Previous reports investigating mechanisms of secondary resistance to nutlin in neuroblastoma and osteosarcoma cell lines suggested that exposure to nutlin induces the emergence of TP53-mutated clones [41, 42]. Whether these mutations appeared de novo in the secondary resistant cells or were already present in a minority of clones was not known. Using deep sequencing, our results suggest that nutlin favors the occurrence of TP53 mutations in initially TP53 wild-type cells.

Besides TP53 mutations, our data identified a subset of other gene mutations that were positively selected in secondary resistant cells, many of which have been previously involved in the control of cell growth or apoptosis. Some of them may represent ‘passenger’ mutations or false-positives, but some are likely to contribute to resistance to RG7388. We highlight two examples here. In IB128 resistant cells, we observed a significant increase in the abundance of an activating mutation in *N-RAS* in the IB128 resistant cells. Activation of the MAPK/ERK kinase (MEK)1/2 kinase pathway has been shown to impair TP53-dependent apoptosis in U20S cells treated with nutlin [43]. Recent data showed that combining the MDM2-p53 protein-protein interaction inhibitor SAR405838 and the MEK inhibitor pimasertib resulted in synergic anti-tumor activity in several K-RAS, N-RAS and B-RAF mutant tumor models [44]. The possibility that combining a MEK inhibitor with nutlin could overcome secondary resistance deserves further investigation. In IB111 resistant cells, we observed an increase in abundance of a missense mutation of the *ECT2* gene, the expression of which has been shown to be normally down-regulated by Nutlin in a TP53-dependent manner [45]. Inactivation of *ECT2* was shown to be sufficient to prevent cell death induced by ionizing radiation underlying its potential important role in resistance to cancer therapy resistance [46].

We have also found that secondary resistance to RG7388 was associated with few acquired ploidy variations. This observation fits with the results of a previous study showing that instable aneuploidy is associated with acquired drug resistance and that nutlin treatment of U2OS osteosarcoma cells and HCT116 colon cancer cells can result in the emergence of tetraploid cells [47]. Here we have extended these previous observations, using deep sequencing, demonstrating that the majority of allelic imbalances were shared by the parental and resistant cells. Altered intrinsic tumor sensitivity to RG7388 was associated with only a few more aberrations. Some of them targeted genomic regions including genes that are directly linked to nutlin activity such as *MDM2* (12q13-15) for IB111 resistant cells or TP53 (17p) for the IB128 resistant cells. This implies that some ancestral cells that carried this complement of ploidy aberrations emerged under RG7388 selection pressure. These additional ploidy aberrations demarcate the split between fully clonal versus subclonal ploidy variations.

It was recently observed that tumor cells vary in terms of their apoptotic activity potential, and that alteration of the apoptotic machinery is a cause of chemoresistance and oncogenic transformation [48–49] By comparing gene expression of parental and secondary resistant cells, we observed in IB111 resistant cells a strong down-regulation of genes encoding for BH3-only factors such as BIM, BMF and PUMA. Interestingly, down-regulation of these genes has been associated with resistance to targeted therapies such as imatinib in bcr-abl+ leukemia cells [50] or vemurafenib in human melanoma cells [51]. Besides being a pro-apoptotic molecule, BMF is also an important regulator of autophagy [52]. Since autophagy has been associated with resistance to radiation, chemotherapy, and targeted agents [30–39], we decided to investigate whether secondary resistance to RG7388 was associated with autophagy induction in STS cell lines. Interestingly, we found that IB111 resistant cells showed pronounced autophagy whereas no autophagy was observed in IB115 and IB128 resistant cells. Moreover, we have demonstrated that autophagy inhibition significantly enhanced RG7388-induced cell death in IB111 resistant cells. We report here that down-regulation of genes encoding pro-apoptotic molecules and autophagy induction are important mechanisms involved in secondary resistance to nutlin. These observations indicate that in the presence of nutlin autophagy can promote cell adaptation and survival.

In conclusion, our results demonstrate that secondary resistance of STS cells to nutlin involves multiple and complex mechanisms and that the interplay between these mechanisms warrants extensive investigation. As such events do not appear to impact sensitivity to cytotoxic agents commonly given to STS patients, therapeutic strategies preventing their emergence should be designed. One of them could be administering nutlin for a restricted period, alternating with other drugs and then restarting, irrespective of disease progression status in the period between treatments.

## Supporting Information

S1 Fig (DOCX)

S2 Fig (DOCX)

S3 Fig (DOCX)

S1 Methods (DOCX)

S1 Table (DOCX)

S2 Table (DOCX)
