# Supplementary material for: Heterogeneous Mechanisms of Secondary Resistance and Clonal Selection in Sarcoma during Treatment with Nutlin
Source: PLoS One. 2015 Oct 1;10(10):e0137794. doi: 10.1371/journal.pone.0137794 (PMC4591276; doi:10.1371/journal.pone.0137794)
Supplement: S1 Fig — (DOCX) [file pone.0137794.s001.docx]

**
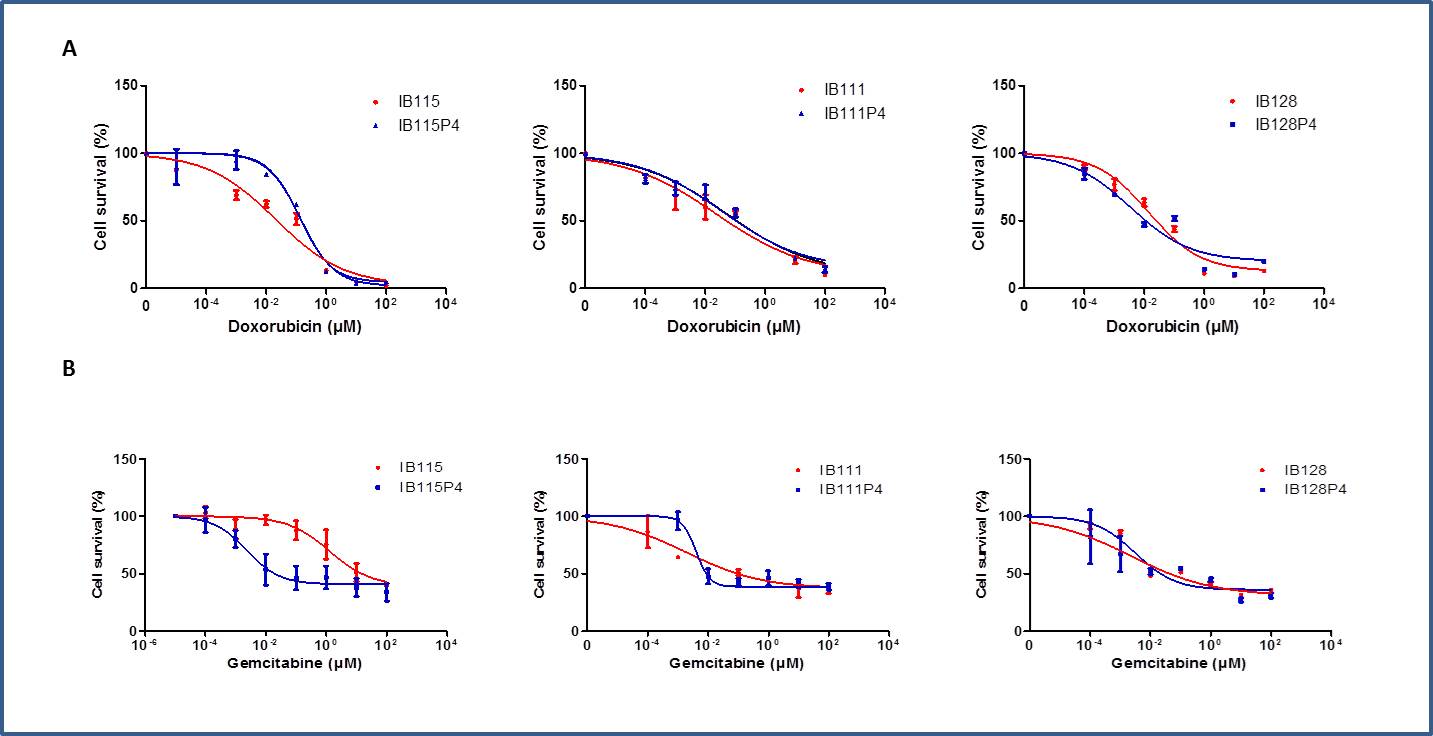
**

**Supplementary Figure 1.** Antiproliferative activity of doxorubicin (A) and gemcitabine (B) in the three sensitive and resistant IB111, IB115 and IB128 cell lines after 72h of treatment measured by MTT
