# Supplementary material for: Heterogeneous Mechanisms of Secondary Resistance and Clonal Selection in Sarcoma during Treatment with Nutlin
Source: PLoS One. 2015 Oct 1;10(10):e0137794. doi: 10.1371/journal.pone.0137794 (PMC4591276; doi:10.1371/journal.pone.0137794)
Supplement: S2 Fig — (DOCX) [file pone.0137794.s002.docx]

**
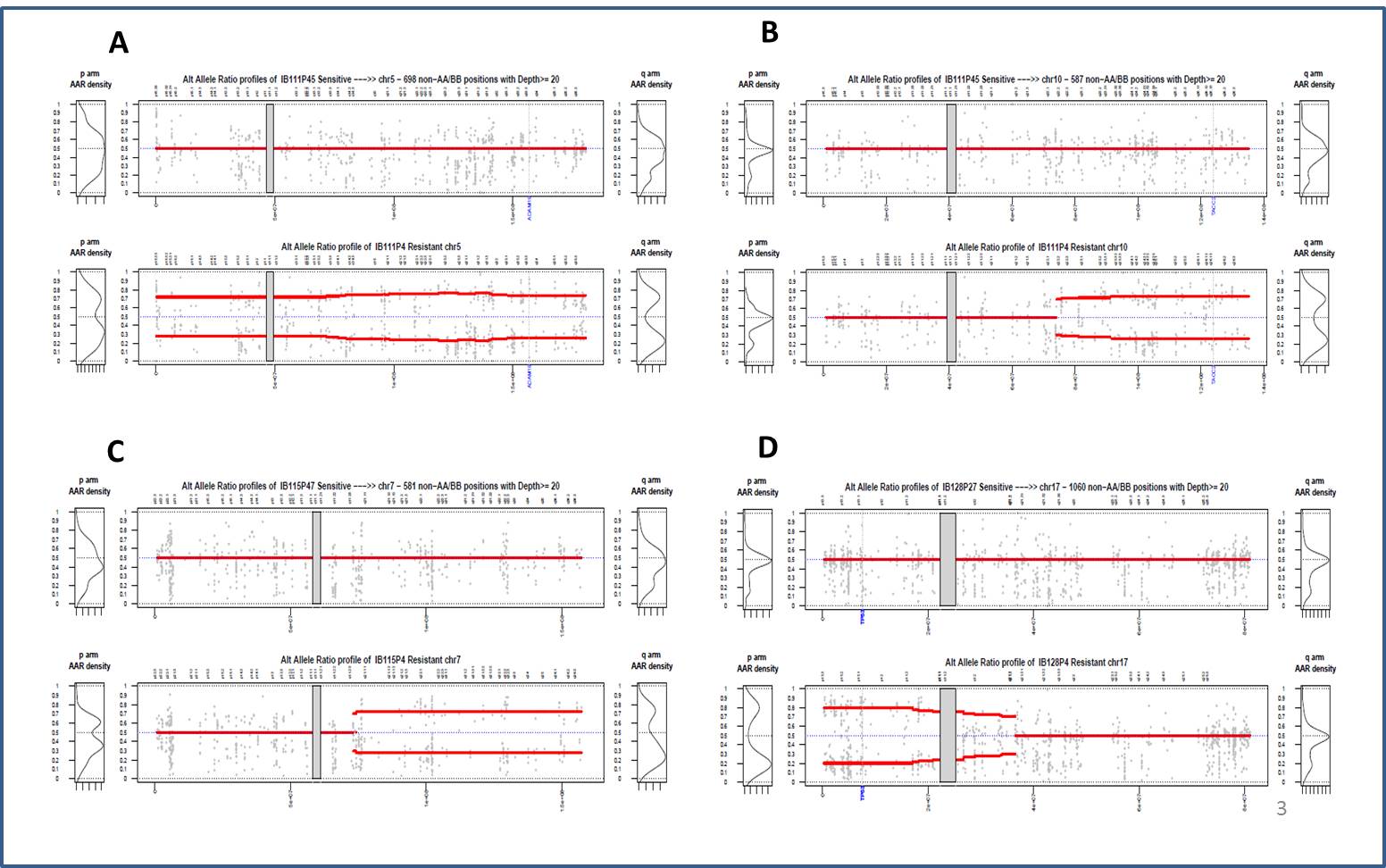
**

**Supplementary Figure 2. Genomic ploidies characterizing STS cells secondary resistant to RG7388 clones (Top plots: B-Allele Frequency [BAF] of sensitive clones, bottom plots BAF of resistant clones).** A, B: IB111, chr5q tetra-ploidy and chr10q22.2, tetra-ploidy in secondary resistant cells; C: IB115, chr7q tetra-ploidy in secondary resistant cells; D: IB128, chr17p penta-ploidy in secondary resistant cells.
