# Supplementary material for: Heterogeneous Mechanisms of Secondary Resistance and Clonal Selection in Sarcoma during Treatment with Nutlin
Source: PLoS One. 2015 Oct 1;10(10):e0137794. doi: 10.1371/journal.pone.0137794 (PMC4591276; doi:10.1371/journal.pone.0137794)
Supplement: S3 Fig — (DOCX) [file pone.0137794.s003.docx]

**
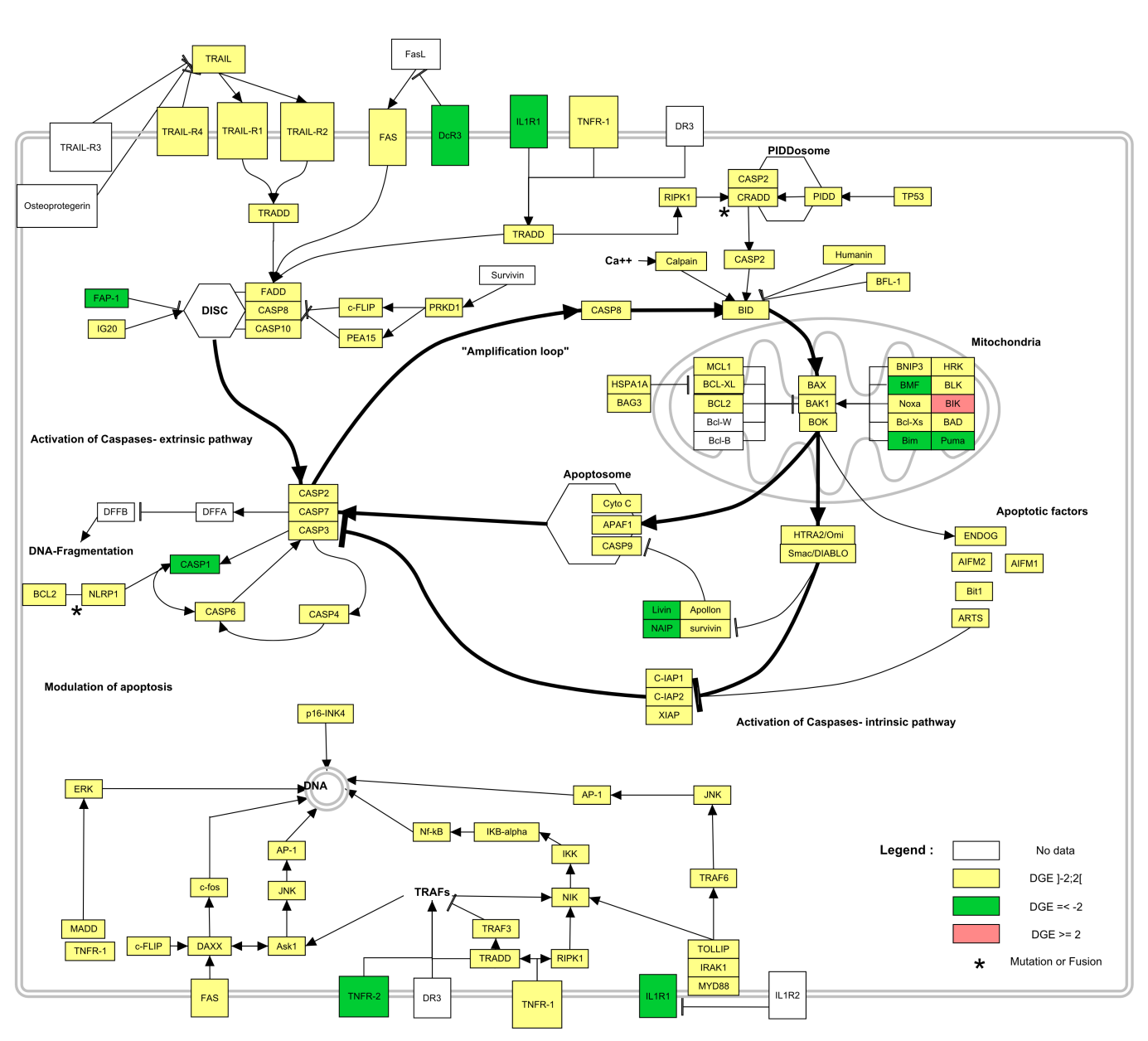
**

**Supplementary Figure 3.** Deregulation of the expression of genes involved in the apoptosis pathway (IB111/IB111P4 cell lines). WikiPathways - Apoptosis Modulation and Signaling (Homo sapiens): http://www.wikipathways.org/index.php/Pathway:WP1772 (66). DGE: differential gene expression
