# Supplementary material for: Heterogeneous Mechanisms of Secondary Resistance and Clonal Selection in Sarcoma during Treatment with Nutlin
Source: PLoS One. 2015 Oct 1;10(10):e0137794. doi: 10.1371/journal.pone.0137794 (PMC4591276; doi:10.1371/journal.pone.0137794)
Supplement: S1 Methods — (DOCX) [file pone.0137794.s004.docx]

**SUPPLEMENTARY METHODS**

*PGM Sequencing*

DNA extraction was performed with Qiamp DNA mini kit (Qiagen, Courtaboeuf, France) as described by the manufacturer. The purity of the genomic DNA was measured with a Nano-Drop 1000 apparatus (NanoDrop Products), and the quantity was estimated by a fluorescence-based method using a Qubit double-stranded DNA BR assay kit and a Qubit fluorometer (Life Technologies, Germany) according to the manufacturer’s instructions.

Sequence libraries were prepared from 10ng of DNA according to the manufacturer’s protocol with the Ion AmpliSeq^TM^ Library Kit 2.0 and the Ion AmpliSeq TM Custom panel design TP53 exons (Life Technologies, NY, USA). Each library was barcoded with the Ion Xpress^TM^ Barcode Adapters 1–16 Kit (LifeTechnologies, NY, USA). Each library was normalized to 100pM after quantification on the 2200 Tapestation with the high sensitivity D1000 Screen tape assay (Agilent technologies, Santa Rosa, California, USA). Then, emulsion PCR was performed from the pooled libraries on the IonOneTouch2^TM^ with the Ion OneTouch^TM^ 200 Template KitV2. After enrichment of the Ion Sphere Particles on the Ion Onetouch ES^TM^, template libraries were sequenced on the Ion Torrent PGM^TM^ with the Ion Torrent PGM Sequencing 200 Kit v2 on a Ion 318Chip Kit v2 (Life Technologies, NY, USA).

*Genomic ploidy estimation*

We exploited RnaSeq to provide an estimate of the ploidy of the tumor samples in large genomic regions. The term ploidy is used here to define the total number of parental alleles in a genomic region. Under the hypothesis that a gene is transcribed 5’ to 3’ on both parental alleles (bi-allelic) or one single allele (mono-allelic), then the Minimal Ploidy of a genomic region will be estimated by the B Allele Frequency or BAF and the related genotypes of consecutive genetic variants observed in genomic coding regions. The following table reports the BAF thresholds, the related genotype scenarios and the associated Minimal Ploidies (indicated by an integer followed by P).

| **BAF** | **GENOTYPES** | **Minimal Ploidy** |
| --- | --- | --- |
| 0.5 | A/B, AA/BB, AAA/BBB | 2P |
| 0.33 | AA/B, AAAA/BB, AAAAAA/BBB … | 3P |
| 0.66 | A/BB, AA/BBBB, AAA/BBBBBB | 3P |
| 0.25 | AAA/B, AAAAAA/BB, AAAAAAAAA/BBB … | 4P |
| 0.75 | A/BBB, AA/BBBBBB, AAA/BBBBBBBBB … | 4P |
| 0.20 | AAAA/B, AAAAAAAA/BB, AAAAAAAAAAAA/BBB … | 5P |
| 0.80 | A/BBBB, AA/BBBBBBBB, AAA/BBBBBBBBBBBB … | 5P |

All genotype scenarios with the same BAF threshold have a number of alleles that are multiples of the Minimal Ploidy. The term Minimal is used to indicate the smallest number of alleles giving the same BAF threshold. (Ploidy is used as a shortcut in the text). In case of bi-allelic expression, the estimation of ploidy is similar to the one obtained via DNA sequencing. If a gene is instead mono-allelic expressed, we cannot distinguish genomic loss-of-heterozygosis and mono-allelic expression. An additional limitation is that if the expression of just one allele is observed in a large genomic region covering several genes, we cannot distinguish between a genomic LOH and a uniparental disomy region.

To detect Minimal Ploidy profiles for each chromosome we applied an approach similar to the one used by Popova et al. (1):

- we extracted the positions identified as non-homozygous,

- we mirrored the BAF with respect to the 0.5 ratio to determine the mBAF (mirrored BAF),

- and we then used the package Runmed from the R library Stats to produce the running median of the mBAF (rolling window of 301 markers).

In order to work out when the moving average is informative of imbalanced ploidy, we used a sanity check cut-off of 0.6, corresponding to the central binomial 90% confidence interval of the heterozygous frequency 0.5 for a total number of 50 reads aligned to a given position. When the running median was above the cut-off, a region of imbalanced ploidy was called.

All regions with running median falling below the cut-off were called as balanced ploidy, hence Minimal Ploidy of 2. The start and end coordinates of each region were estimated applying the algorithm CBS (2).

*NGS Differential Gene Expression (DGE) pipeline*

The RnaSeq Differential Gene Expression between two NGS samples was calculated using the following methodology implemented in R language. Briefly, the number of aligned reads falling into each gene were counted using HTSeq (3).

The length of each gene was calculated using DEXSeq (perl script as in (4,5). Then the raw transcript-counts for each gene were calculated as:

Transcripts(gene) = Read_Counts(gene) * Number_Bases_in_PE_read / Length(gene)

The nb of transcripts per gene was then normalized as follows under the hypothesis that the library size was the only biasing factor and in the case of two samples comparison. Let A, B be the two samples; let Sum(A) and Sum(B) be their library sizes in terms of transcripts counts

Sum(A) = sum Transcripts(gene,A)

Sum(B) = sum Transcripts(gene,B)

Let A be the smaller library size. We then derive the Adjusted Transcript counts of B to build the null hypothesis that B has the same library size as A. To do so we define the total difference in libraries as:

Total_DIFF = Sum(B) - Sum(A)

and the percentage contribution of each gene to B library as:

Percent(gene,B) = Trancripts(Gene) / sum(B)

We then calculate the absolute contribution of each gene to Total_DIFF by:

ContribDIFF(gene,B)=Perc(gene,B)*Total_DIFF

Using the above quantities, we adjust the transcript counts of each gene in B by:

ADJ_Transcripts(gene,B) = Transcripts(gene,B) - ContribDIFF(gene,B)

In order to compare the adjusted transcript count of a gene in B to the (un-adjusted) transcript counts of the same gene in A, we apply the following Statistical test for Counts, under the Null Hypothesis that the two libraries are equally distributed. Let ADJ_B and A be two samples having the same library distribution. We compare the counts ADJ_Transcripts(gene,B), given the total Sum(ADJ_B)), to the counts Transcripts(gene,A), given Sum(A), using a Relative Risk test.

Let x1 = ADJ_Transcripts(gene,B), x2 = Sum(ADJ_B) - x1, x3 = Transcripts(gene,A), x4 = Sum(A) - x3. Let rx1 = x1/(x1 + x2) be the risk of the gene in B, rx3 = x3/(x3 + x4) the risk of the gene in A, rr=rx1/rx3 be the relative risk and lnrr=log(rr) be the log relative risk. Let selnrr = sqrt(1/x1 - 1/(x1+x2) + 1/x3 - 1/(x3+x4)) be the standard error of the log-relative risk according to Delta Method (6). According to the Delta method when sample size is large (i.e. x1+x2 and x3+x4 are large), the test of the log relative risk is approximately the test of a normal variable having mean equal to the log-relative-risk and standard error equal to the standard error of the log relative risk. The p-values of the test are directly obtained from the cumulative probability of the normal distribution.

**Supplementary References:**

1. [Popova T](http://www.ncbi.nlm.nih.gov/pubmed?term=Popova%20T%5BAuthor%5D&cauthor=true&cauthor_uid=19903341), [Manié E](http://www.ncbi.nlm.nih.gov/pubmed?term=Mani%C3%A9%20E%5BAuthor%5D&cauthor=true&cauthor_uid=19903341), [Stoppa-Lyonnet D](http://www.ncbi.nlm.nih.gov/pubmed?term=Stoppa-Lyonnet%20D%5BAuthor%5D&cauthor=true&cauthor_uid=19903341), [Rigaill G](http://www.ncbi.nlm.nih.gov/pubmed?term=Rigaill%20G%5BAuthor%5D&cauthor=true&cauthor_uid=19903341), [Barillot E](http://www.ncbi.nlm.nih.gov/pubmed?term=Barillot%20E%5BAuthor%5D&cauthor=true&cauthor_uid=19903341), [Stern MH](http://www.ncbi.nlm.nih.gov/pubmed?term=Stern%20MH%5BAuthor%5D&cauthor=true&cauthor_uid=19903341)., Genome Alteration Print (GAP): a tool to visualize and mine complex cancer genomic profiles obtained by SNP arrays. Genome Biol. 2009;10:R128.
2. Venkatraman, ES, Olshen AB. A faster circular binary segmentation algorithm for the analysis of array cgh data. Bioinformatics 2007;23:657-63.
3. R Core Team R: A language and environment for statistical computing. R Foundation for Statistical Computing, Vienna, Austria. 2013 URL: <http://www.R-project.org/> ).
4. Anders S, Pyl PT, Huber W. HTSeq — A Python framework to work with high-throughput sequencing data [Bioinformatics.](http://www.ncbi.nlm.nih.gov/pubmed/?term=HTSeq+%E2%80%94+A+Python+framework+to+work+with+high-throughput+sequencing+data) 2014, In Press.
5. Anders S, Reyes A, Huber W. Detecting differential usage of exons from RNA-seq data Genome Res. 2012;10: 2008-2017.
6. Casella G, Berger RL. Statistical Inference, 2nd ed, 2002.
