# Supplementary material for: Heterogeneous Mechanisms of Secondary Resistance and Clonal Selection in Sarcoma during Treatment with Nutlin
Source: PLoS One. 2015 Oct 1;10(10):e0137794. doi: 10.1371/journal.pone.0137794 (PMC4591276; doi:10.1371/journal.pone.0137794)
Supplement: S1 Table — (DOCX) [file pone.0137794.s005.docx]

| **Cell line ID** | **Histological subtype** | **TP53 mutational status*** | **MDM2 amplification status** |
| --- | --- | --- | --- |
| IB111 | Dedifferentiated liposarcoma | Wild-type | Amplified |
| IB112 | Leiomyosarcoma | Null | Normal |
| IB114 | Myxofibrosarcoma | Wild-type | Gail |
| IB115 | Dedifferentiated liposarcoma | Wild-type | Amplified |
| IB118 | Leiomyosarcoma | P278L | Normal |
| IB126 | Synovial Sarcoma | Wild-type | Gain |
| IB128 | Extra-skeletal osteosarcoma | Wild-type | Normal |
| IB130 | Pleomorphic Liposarcoma | P278L | Deleted |
| IB133 | Leiomyosarcoma | Exon 2-3 deleted | Deleted |
| IB134 | Leiomyosarcoma | S215R | Gain |
| IB136 | Leiomyosarcoma | Null | Gain |

**Supplementary Table 1.** Panel of soft-tissue sarcoma cell lines. *Sanger sequencing
