# Supplementary material for: Heterogeneous Mechanisms of Secondary Resistance and Clonal Selection in Sarcoma during Treatment with Nutlin
Source: PLoS One. 2015 Oct 1;10(10):e0137794. doi: 10.1371/journal.pone.0137794 (PMC4591276; doi:10.1371/journal.pone.0137794)
Supplement: S2 Table — (DOCX) [file pone.0137794.s006.docx]

**Supplementary Table 2.** List of genes differentially expressed in parental IB111, IB115 and IB128 and their resistant counterparts (with fold change and p value)
